# Anxiety Reaction in Children During Cast Removal using Oscillating Saw versus Cast Shear - A Randomised, Prospective Trial

**DOI:** 10.5704/MOJ.2107.018

**Published:** 2021-07

**Authors:** NA Mohamed-Zain, K Jamil, R Penafort, A Singh, S Ibrahim, AH Abdul-Rashid

**Affiliations:** 1 Department of Orthopaedics and Traumatology, Universiti Kebangsaan Malaysia, Kuala Lumpur, Malaysia; 2 Department of Orthopaedics, KPJ Damansara Specialist Centre, Kuala Lumpur, Malaysia; 3 Department of Orthopaedics and Traumatology, Tengku Ampuan Rahimah Hospital, Klang, Malaysia

**Keywords:** anxiety, cast removal, oscillating saw, cast shear

## Abstract

**Introduction:**

To compare the anxiety levels demonstrated by children during cast removal procedure between oscillating saw vs cast shear methods.

**Material and methods:**

A randomised prospective study of 102 children (mean age 8.3 ± 3.5 years) with fractures involving upper or lower limbs. Children undergoing removal of cast were divided into 2 groups; either by an oscillating saw or a cast cutting shear. The level of anxiety was assessed by recording the heart rate with a portable fingertip pulse oximeter before, during and after removal of the cast. Objective assessment was performed by documenting the fear level on Children’s Fear Scale (CFS).

**Results:**

There was a significant increase in the heart rate of children during cast removal while using the oscillating saw compared to cast shear (p<0.05). The noise level produced by the saw exceeded 80 dB (mean 103.3 dB). The fear level was significantly lower in the cast shear group (p<0.05).

**Conclusion:**

The noise produced by the oscillating saw was associated with an increased anxiety level in children undergoing cast removal. Cast shear is a simple and inexpensive instrument that can be used for cast removal in overly anxious children.

## Introduction

Approximately one-third of children suffer fractures by the age of 16 years, with increasing incidence until the age of 12 years in girls and 14 years in boys (1). Ninety percent of paediatric fractures are treated non-operatively, in which casting is the most preferred method for fracture immobilisation^1,2^. Anxiety caused by the injury causing the fracture can affect the child not only emotionally but physically. Although parental reports dismissed children’s display of anxiety after an accident as mild and short lived, a third of them was found to suffer from post-traumatic stress disorder^3^. Furthermore, the removal of a plaster cast with an oscillating saw which is accompanied by a high-frequency noise may cause more anxiety and is potentially harmful to the children^4-6^. An extreme example would be death that was reported in a child after cast removal with the oscillating saw^4^. In this child with a pre-existing heart condition, extreme anxiety possibly caused the fatal cardiac arrhythmia^4^.

The noise from a cast saw and fear of its use are the common sources of anxiety from a child’s perspective^7^. It has been reported that the peak noise level of cast room clinics far exceeded the recommended limits of 85 decibels (dB), which can be hazardous to both adults and children^5^. In contrast, a cast shear can be as effective as a saw for cast removal with hardly any noise.

To the best of our knowledge, the comparison of children’s anxiety level during removal of cast with either the oscillating saw or cast shear has not been investigated. We conducted a prospective randomised study to compare the level of anxiety in this two groups, as well as measuring the cast room noise level during cast removal procedures.

## Materials and Methods

This was a randomised prospective study of children with closed fractures involving the upper limbs or lower limbs. The study was approved by the national medical research register ethics committee review board. The children were divided into two groups; removal of cast by an oscillating saw [GP 104, AESCULAP, Germany] or by a cast cutting shear [AE-LX574R, STILLE-AESCULAP, Germany]. Randomisation was done via simple randomisation method into two treatment groups. Allocation of participants using computer-generated list of numbers (even and odd) in random sequence was performed. They were put into a sequentially numbered sealed envelope by a study coordinator who was not involved in the patient’s management. Once enrolled, the envelope would be opened by participants just before the procedure. The study coordinator would then assign the subjects into their respective groups. The study was done at a tertiary hospital with a high patient load from June 2013 until May 2014.

The inclusion criteria for this study were children aged 1 to 13 years old who had traumatic fractures of either the upper limbs or lower limbs treated with plaster casts and had no previous fractures. Children who had pathological fractures, multiple fractures involving more than one limb, polytrauma and with underlying cardiac disease were excluded.

**Fig. 1:**
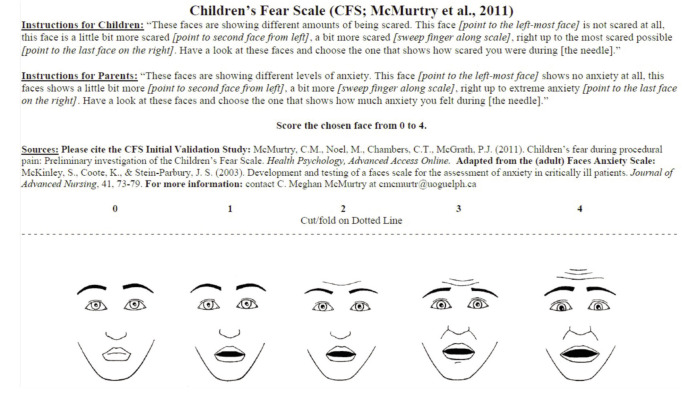
Children’s fear scale.

The level of anxiety was assessed by recording the heart rate with a portable fingertip pulse oximeter placed on the index finger of the child. The heart rate was monitored for 1 minute at different intervals; before, during and immediately after cast removal. The peak heart rate of each session was documented. At the same time, the oxygen saturation during the procedure was also recorded. The cast room noise level was measured (in dB) using sound level meter [AR824, Smart Sensor, China]. After completion of the procedure, the children were asked to indicate their fear level related to the cast removal on the Children’s Fear Scale^8^, which is an objective assessment of the level of anxiety (Fig. 1).

Obtaining consents, pulse oximeter placements, and heart rate recordings were performed by a single investigator. Both the investigator and patient were not blinded for the treatment method. Pulse oximeter probes were placed on the children in an isolated waiting room and left on the fingers up until 1 minute after the procedure. Parents were given full explanation of the use of the instruments and stayed near their child during the procedure in all cases. They were allowed to assist their children in marking the fear scale level, when necessary.

The data were recorded in 'Microsoft excel' file and stored in a computer database only accessible to the investigators. Data are stored in a de-identified format identifiable only by study number, with codes accessible only to investigators to ensure subject confidentiality and privacy.

Sample size was determined based on a previous study by Katz *et al*^4^, who investigated children wearing noise protectors during cast removal with saw had a mean heart rate of 103±8.14 beats/minute. We hypothesised that children who have their cast removed with cast shear would have a similar outcome or at most 5% increase of the heart rate compared to wearing noise protectors during saw-cast removal. Utilising the sample size calculator, 94 patients was adequate to represent the 5% difference with 80% power and level of significance of 0.05. We chose to recruit a total of 102 subjects overall to account for 10% who might be excluded and achieve equal allocation. Statistical analysis was performed using student's t-test and Chi-square test for continuous and categorical data, respectively. SPSS [v24, IBM, NY, USA] was used where statistical significance was assumed for p < 0.05.

## Results

**Fig. 2:**
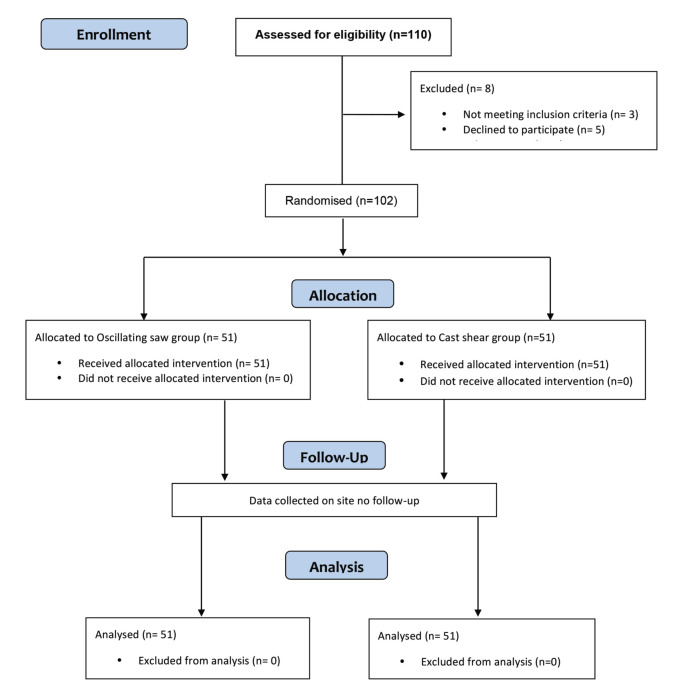
The study flow diagram

From a total of 110 patients, eight subjects were excluded (3 revealed history of previous fractures, 5 declined to participate). A total of 102 subjects were randomised to either cast shear (N=51) or oscillating saw (N=51) groups for the study (Fig. 2). Table I shows the subjects characteristics. There were no significant differences seen between the groups in terms of age (p=0.06) and gender (0.55). Plaster of Paris casts were removed for upper limb fractures from 56 subjects (29 both radius and ulna, 12 radius and 15 ulna), while the other 46 subjects had lower limb fractures (9 femur, 37 tibia). Comparison between the groups revealed that the oscillating saw group comprised more lower limb fractures (57%), whilst higher distribution of upper limb fractures was seen in the cast shear group (66.7%), p<0.05.

**Table I:** The characteristics of the study participants. Age is expressed as mean ± standard deviation while gender and fracture location are presented as total number (percentage)

	Oscillating saw (N=51)	Cast shear (N=51)	P value
Age in years (mean ± SD)	8.9 ± 3.1	7.7 ± 3.7	0.06
Gender N (%)			
Male	30 (58.8%)	27 (52.9%)	0.55
Female	21 (41.2%)	24 (47.1%)	
Fracture location N (%)			
Upper limb	22 (43.1%)	34 (66.7%)	<0.05
Lower limb	29 (56.9%)	17 (33.3%)	

Table II summarises the results of the study. In the oscillating saw group, the mean heart rate of the children was 97.3±15.4 beats per minute (bpm) before removal of cast and 109.4±16.4 bpm during the procedure, a mean increase of 12.14±13.53 bpm (p<0.05). In the cast shear group, the mean heart rate of the children was 93.1±17.4 bpm before removal of cast and was reduced (mean heart rate 89.3±15.9 bpm) during the procedure. There is no statistical difference in comparison of pre-removal heart rate between the groups (p=0.20). When divided into sub-groups according to age and location of cast, the oscillating saw group consistently recorded a higher mean heart rate in comparison to cast shear group (p<0.05) (Table III).

**Table II:** Comparison of the children’s heart rate, level of anxiety and cast room noise level between the oscillating saw versus cast shear group

	Oscillating saw (N=51)	Cast shear (N=51)	p value
Heart rate, beats/min (mean±SD)			
Before procedure	97.29±15.40	93.10±17.40	0.20
During procedure	109.43±16.40	89.25±15.90	<0.05
After procedure	103.82±15.02	89.82±19.93	<0.05
Mean noise level, dB (mean±SD)	103.3±19.10	72.2±7.52	<0.05
Children’s Fear Scale (count)			
Scale 1	0	3	<0.05
Scale 2	6	18	
Scale 3	14	26	
Scale 4	31	4	

**Table III:** Comparison of heart rate between oscillating saw and cast shear group according to different age group and location of cast, during the procedure

	Age below 11 years old (N=66)	Age 11 and above (N=36)	Upper limb cast (N=56)	Lower limb cast (N=46)
Heart rate, beats/min (mean±SD)				
Oscillating saw	112.63±15.12	104.86±17.45	113.55±16.00	106.31±16.29
Cast shear	93.03±15.63	80.20±13.07	90.26±16.83	87.24±14.20
p value	<0.05	<0.05	<0.05	<0.05

**Fig. 3:**
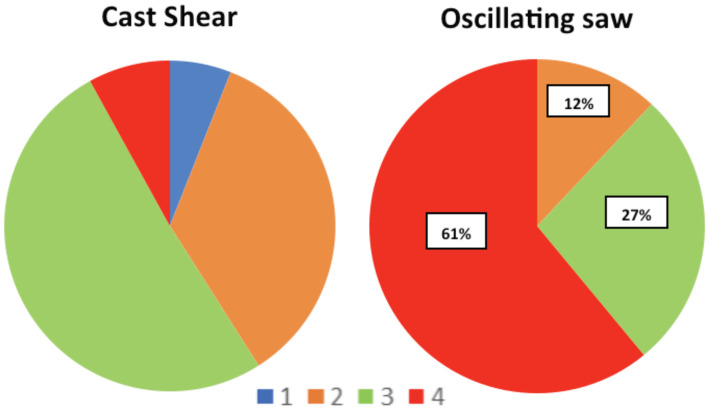
Comparison of Children’s Fear Scale in cast shear and oscillating saw group. Score 1 to 4, represented by the respective colours. Number of patients expressed in percentages (%).

In the anxiety level measured using the Children’s Fear Scale, the cast shear group showed that 51% marked scale 3 while only 8% marked the highest scale which was scale 4. Three children indicated scale 1 (6%), and the rest scale 2 (35%). In the oscillating saw group however, 61% marked scale 4 and the lowest fear scale indicated by the children was scale 2 (Fig. 3). The fear scale level in the cast shear group was significantly lower than the oscillating saw group (p<0.05).

There was a weak correlation between the peak heart rate during the cast removal procedure and the level of anxiety indicated by the Children’s Fear Scale (r=0.3, p<0.05). However, there was no significant difference between oxygen saturation in both groups with a mean level of 98.6%.

**Fig. 4:**
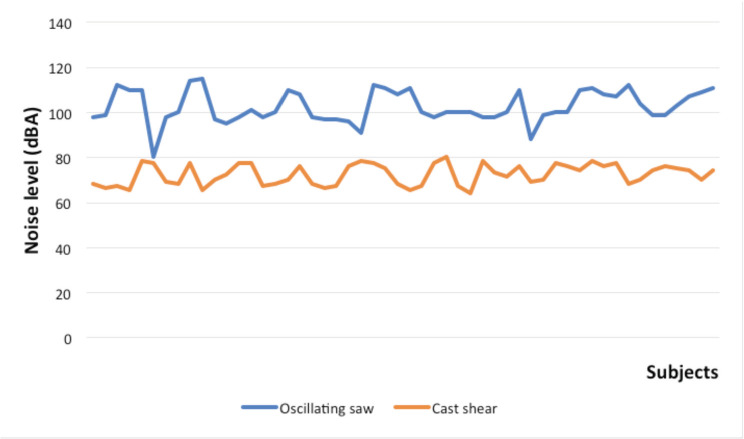
Cast room noise level during removal of cast in oscillating saw group and cast shear group. Y-axis: noise level measurements in dBA. X-axis: individual subjects.

Noise level measurement from the cast room during cast removal using the oscillating saw showed a level ranging from 80 to 115.2 dB with the mean of 103.3 dB. In the cast shear group, the noise level was much lower (range from 65.6-79.4 dB) with the mean of 72.2 dB (p<0.05) (Fig. 4). No complication was encountered during the cast removal procedure in both groups.

## Discussion

Plaster cast removal is one of the outpatient procedures that is known to cause anxiety in children. It is mainly due to the noise of the oscillating saw, and many studies have proposed the usage of a hearing protection device during the procedure^4,6,7^. Other factors, such as damage to the skin or the heat generated by the saw blade, may contribute to the anxiety as well^4^. A cast shear, a simple instrument available at most orthopaedic clinics can be used for the same purpose to reduce the noise level.

The heart rate has been used as an indicator of anxiety as anxiety causes increased sympathetic activity, which in turn increases conduction velocity and decreases the cardiac refractory period^9^. Katz *et al*^4^ evaluated the usefulness of hearing protectors to reduce anxiety during plaster cast removal. Their study showed that the mean increase of heart rate in the group without hearing protectors is higher compared to the children with hearing protectors. Carmichael *et al*^7^ concurred with Katz’s study, but found 25% of the children in their study did not tolerate wearing hearing protectors^7^. Furthermore, not all centres provide this device for their patients. Other methods of managing anxiety might be more suitable.

Some researchers used ‘distraction’ methods to control anxiety in children during cast room procedures. Liu and colleagues found that lullaby music decreased anxiety among paediatric patients in the cast room^10^. Lower heart rates were detected among children in the music therapy group during outpatient procedures. In another study, watching videos on a portable device were shown to have a significant effect on reducing heart rate in children before cast procedures^11^. More recently, a large clinical trial investigating therapeutic play intervention in children before cast removal procedures revealed that it was effective in reducing anxiety levels^12^. In this trial, a combination of subjective and objective assessments comprising heart rate measurements and various questionnaires were utilised to evaluate anxiety.

Similarly, our study showed that using cast shear for cast removal was associated with a reduced heart rate in the patients, leading to a reduction in anxiety compared with using an oscillating saw. Pre-removal heart rate was not significantly different between the groups, indicating that the children were more anxious after being exposed to the noise in the cast saw group. Comparing the two groups according to different age groups and location of cast also revealed the heart rate was consistently higher in the oscillating saw group. Hence, older children (11 to 13 years in this study) were similarly affected as their younger counterparts. We utilised the validated Children’s Fear Scale^8^, a pictorial face scale to quantify the level of anxiety and found a positive correlation with the heart rate. An increased heart rate was associated with a higher level of anxiety on the Children’s Fear Scale.

A study in Canada suggested that the noise level in the cast room can exceed the safety limit and is potentially hazardous to patients including the paediatric population^5^. A mean of 103.3 dB for sound intensity in our study was very loud, equivalent to a sound produced by a farm machinery^13^. Children may be more vulnerable in acquiring noise-induced hearing impairment than adults and instantaneous high peak noise level of more than 120 dB may lead to auditory damage^14^. A long-term exposure (>8 hours) to noise levels of more than 85 dB may also induce hearing impairment^5,13^. Recommended noise exposure limits may differ between national health organisations. A study by Post *et al* however, revealed that the high noise level in the cast room might not be clinically relevant^13^. They studied three different types of oscillating saws including a ‘Quiet Cast Removal System’ specifically designed for noise reduction. Comparing all three, even at the highest peak sound level produced (99.9 dB) with the distance of 6 inches from the ear, a child needs to be exposed to it for more than 15 minutes to surpass the accepted National Institute of Occupational Safety and Health (NIOSH) recommendation^13^.

Limitation of the present study is that we were unable to perform a blinded experiment due to the nature of the study and working conditions in the hospital therefore the element of observer bias can’t be completely eliminated. We utilised a simple randomisation method and did not stratify the subjects according to specific variables such as age, gender and weight of subjects, or site of fracture. These variables could have impacted our results. Although, our subgroup analysis showed oscillating saw group produced higher mean heart rate than cast shear, independent of age or site of fracture. Furthermore, we did not specifically look into the time taken for the removal of cast, but we only include a single limb procedure, so prolonged exposure beyond 5 minutes is very unlikely. We also did not investigate other factors such as the saw distance to the patient’s ear or size of the cast room, which may influence the interpretation of our results. However, the location of the cast (either upper limb or lower limb) might represent the saw-to-ear distance and we did not find any difference between the two.

## Conclusion

We found that the noise produced by the oscillating saw in our study was associated with an increased heart rate and anxiety level in children undergoing cast removal. The noise level measured was very high, although the potential effect of noise-induced hearing loss couldn’t be determined. Current literature suggested that hearing protectors, noise-reducing saws and many other strategies can be used to limit anxiety reaction in children. We propose using a cast shear which eliminates the oscillating saw noise as an option for overly anxious children.

## References

[1] Hamilton T, Hutchings L, Alsousou J, Tutton E, Hodson E, Smith C (2013). The treatment of stable paediatric forearm fractures using a cast that may be removed at home: comparison with traditional management in a randomised controlled trial.. Bone Joint J..

[2] Court-Brown CM, Aitken S, Hamilton TW, Rennie L, Caesar B (2010). Nonoperative fracture treatment in the modern era.. J Trauma..

[3] Stallard P, Velleman R, Baldwin S (1998). Prospective study of post-traumatic stress disorder in children involved in road traffic accidents.. BMJ..

[4] Katz K, Fogelman R, Attias J, Baron E, Soudry M (2001). Anxiety reaction in children during removal of their plaster cast with a saw.. Bone Joint Surg J..

[5] Marsh JP, Jellicoe P, Black B, Monson RC, Clark TA (2011). Noise levels in adult and pediatric orthopedic cast clinics.. Am J Orthop (Belle Mead NJ)..

[6] Wiggins CE, Brown KD. (1996). Hearing protection and cast saw noise.. J South Orthop Assoc..

[7] Carmichael KD, Westmoreland J (2005). Effectiveness of ear protection in reducing anxiety during cast removal in children.. Am J Orthop (Belle Mead NJ)..

[8] McMurtry CM, Noel M, Chambers CT, McGrath PJ (2011). Children's fear during procedural pain: preliminary investigation of the Children's Fear Scale.. Health Psychol..

[9] Hoffmann J, Grimm W, Menz V, Knop U, Maisch B (1996). Heart rate variability and major arrhythmic events in patients with idiopathic dilated cardiomyopathy.. Pacing Clin Electrophysiol..

[10] Liu RW, Mehta P, Fortuna S, Armstrong DG, Cooperman DR, Thompson GH (2007). A randomized prospective study of music therapy for reducing anxiety during cast room procedures.. J Pediatr Orthop..

[11] Ko JS, Whiting Z, Nguyen C, Liu RW, Gilmore A (2016). A Randomized Prospective Study Of The Use Of Ipads In Reducing Anxiety During Cast Room Procedures.. Iowa Orthop J..

[12] Wong CL, Ip WY, Kwok BMC, Choi KC, Ng BKW, Chan CWH (2018). Effects of therapeutic play on children undergoing cast-removal procedures: a randomised controlled trial.. BMJ Open..

[13] Post JM, Switzer KD, Brown DK, Meinzen-Derr J, Dively J, Dunkin BS (2013). Cast saw noise does not reach occupational hazard levels.. J Pediatr Orthop..

[14] Berglund B, Lindvall T, Schwela DH (1999). Guidelines for community noise. Geneva: World Health Organization (WHO). http://https://apps.who.int/iris/handle/10665/66217.

